# Disparities in casemix, acute interventions, discharge destinations and mortality of patients with traumatic brain injury between Europe and India

**DOI:** 10.7189/jogh.14.04227

**Published:** 2024-11-11

**Authors:** Deepak Gupta, Ranjit D Singh, Rick JG Vreeburg, Jeroen TJM van Dijck, Hugo F den Boogert, Kaveri Sharma, Kokkula Praneeth, David B Clarke, Fiona E Lecky, Andrew IR Maas, Virendra Deo Sinha, Godard CW de Ruiter, Wilco C Peul, Thomas A van Essen, CENTER-TBI, Cecilia Åkerlund, Krisztina Amrein, Nada Andelic, Lasse Andreassen, Audny Anke, Anna Antoni, Gérard Audibert, Philippe Azouvi, Maria Luisa Azzolini, Ronald Bartels, Pál Barzó, Romuald Beauvais, Ronny Beer, Bo-Michael Bellander, Antonio Belli, Habib Benali, Maurizio Berardino, Luigi Beretta, Morten Blaabjerg, Peter Bragge, Alexandra Brazinova, Vibeke Brinck, Joanne Brooker, Camilla Brorsson, Andras Buki, Monika Bullinger, Manuel Cabeleira, Alessio Caccioppola, Emiliana Calappi, Maria Rosa Calvi, Peter Cameron, Guillermo Carbayo Lozano, Marco Carbonara, Simona Cavallo, Giorgio Chevallard, Arturo Chieregato, Giuseppe Citerio, Hans Clusmann, Mark Coburn, Jonathan Coles, Jamie D Cooper, Marta Correia, Amra Čović, Nicola Curry, Endre Czeiter, Marek Czosnyka, Claire Dahyot Fizelier, Paul Dark, Helen Dawes, Véronique De Keyser, Vincent Degos, Francesco Della Corte, Hugo den Boogert, Bart Depreitere, Đula Đilvesi, Abhishek Dixit, Emma Donoghue, Jens Dreier, Guy Loup Dulière, Ari Ercole, Patrick Esser, Erzsébet Ezer, Martin Fabricius, Valery L Feigin, Kelly Foks, Shirin Frisvold, Alex Furmanov, Pablo Gagliardo, Damien Galanaud, Dashiell Gantner, Guoyi Gao, Pradeep George, Alexandre Ghuysen, Lelde Giga, Ben Glocker, Jagoš Golubovic, Pedro A Gomez, Johannes Gratz, Benjamin Gravesteijn, Francesca Grossi, Russell L Gruen, Deepak Gupta, Juanita A Haagsma, Iain Haitsma, Raimund Helbok, Eirik Helseth, Lindsay Horton, Jilske Huijben, Peter J. Hutchinson, Bram Jacobs, Stefan Jankowski, Mike Jarrett, Ji Yao Jiang, Faye Johnson, Kelly Jones, Mladen Karan, Angelos G Kolias, Erwin Kompanje, Daniel Kondziella, Evgenios Kornaropoulos, Lars Owe Koskinen, Noémi Kovács, Ana Kowark, Alfonso Lagares, Linda Lanyon, Steven Laureys, Fiona Lecky, Didier Ledoux, Rolf Lefering, Valerie Legrand, Aurelie Lejeune, Leon Levi, Roger Lightfoot, Hester Lingsma, Andrew IR Maas, Ana M Castaño León, Marc Maegele, Marek Majdan, Alex Manara, Geoffrey Manley, Costanza Martino, Hugues Maréchal, Julia Mattern, Catherine McMahon, Béla Melegh, David Menon, Tomas Menovsky, Ana Mikolic, Benoit Misset, Visakh Muraleedharan, Lynnette Murray, Ancuta Negru, David Nelson, Virginia Newcombe, Daan Nieboer, József Nyirádi, Otesile Olubukola, Matej Oresic, Fabrizio Ortolano, Aarno Palotie, Paul M Parizel, Jean François Payen, Natascha Perera, Vincent Perlbarg, Paolo Persona, Wilco Peul, Anna Piippo-Karjalainen, Matti Pirinen, Dana Pisica, Horia Ples, Suzanne Polinder, Inigo Pomposo, Jussi P Posti, Louis Puybasset, Andreea Radoi, Arminas Ragauskas, Rahul Raj, Malinka Rambadagalla, Isabel Retel Helmrich, Jonathan Rhodes, Sylvia Richardson, Sophie Richter, Samuli Ripatti, Saulius Rocka, Cecilie Roe, Olav Roise, Jonathan Rosand, Jeffrey V Rosenfeld, Christina Rosenlund, Guy Rosenthal, Rolf Rossaint, Sandra Rossi, Daniel Rueckert Martin Rusnák, Juan Sahuquillo, Oliver Sakowitz, Renan Sanchez Porras, Janos Sandor, Nadine Schäfer, Silke Schmidt, Herbert Schoechl, Guus Schoonman, Rico Frederik Schou, Elisabeth Schwendenwein, Charlie Sewalt, Ranjit D Singh, Toril Skandsen, Peter Smielewski, Abayomi Sorinola, Emmanuel Stamatakis, Simon Stanworth, Robert Stevens, William Stewart, Ewout W Steyerberg, Nino Stocchetti, Nina Sundström, Riikka Takala, Viktória Tamás, Tomas Tamosuitis, Mark Steven Taylor, Aurore Thibaut, Braden Te Ao, Olli Tenovuo, Alice Theadom, Matt Thomas, Dick Tibboel, Marjolein Timmers, Christos Tolias, Tony Trapani, Cristina Maria Tudora, Andreas Unterberg, Peter Vajkoczy, Shirley Vallance, Egils Valeinis, Zoltán Vámos, Mathieu van der Jagt, Gregory Van der Steen, Joukje van der Naalt, Jeroen TJM van Dijck, Inge AM van Erp, Thomas A van Essen, Wim Van Hecke, Caroline van Heugten, Ernest van Veen, Thijs Vande Vyvere, Roel PJ van Wijk, Alessia Vargiolu, Emmanuel Vega, Kimberley Velt, Jan Verheyden, Paul M Vespa, Anne Vik, Rimantas Vilcinis, Victor Volovici, Nicole von Steinbüchel, Daphne Voormolen, Petar Vulekovic, Kevin KW Wang, Daniel Whitehouse, Eveline Wiegers, Guy Williams, Lindsay Wilson, Stefan Winzeck, Stefan Wolf, Zhihui Yang, Peter Ylén, Alexander Younsi, Frederick A Zeiler, Veronika Zelinkova, Agate Ziverte, Tommaso Zoerle, CINTER-TBI, Deepak Agrawal, Khursheed Alam Khan, Sanjeev Bhoi, Ashish Bindra, Sachin Borkar, Ajay Choudhary, Madhur Choudhary, Shivanand Gamanagatti, Nand Kishore Gora, Deepak Gupta, Amit Gupta, SS Kale, Shweta Kedia, Ashima Nehra, Kokkula Praneeth, Girija Rath, GD Satyarthee, Arul Selvi, BS Sharma, Kaveri Sharma, Rajeev Sharma, Pankaj Kumar Singh, VD Sinha, Sumit Sinha, Vivek Tandon

**Affiliations:** 1 JPN apex Trauma Centre, All India Institute of Medical Sciences, Department of Neurosurgery, New Delhi, India; 2 University Neurosurgical Centre Holland (UNCH), Leiden University Medical Centre, Haaglanden Medical Centre and Haga Teaching Hospital, Department of Neurosurgery, Leiden and The Hague, The Netherlands; 3 Department of Surgery, Division of Neurosurgery, QEll Health Sciences Centre and Dalhousie University, Halifax, Nova Scotia, Canada; 4 Centre for Urgent and Emergency Care Research (CURE), Sheffield Centre for Health and Related Research, School of Population Health, Faculty of Medicine and Health, University of Sheffield, Sheffield, UK; 5 Emergency Department, Salford Royal Hospital, Northern Care Alliance NHS Foundation Trust, UK; 6 Department of Neurosurgery, Antwerp University Hospital, Edegem, Belgium; 7 Department of Translational Neuroscience, Faculty of Medicine and Health Science, University of Antwerp, Antwerp, Belgium; 8 Department of Neurosurgery, Santokba Durlabhji Memorial Hospital cum Medical Research Institute, Jaipur, Rajasthan, India; 9 Department of Neurosurgery, Sawai Man Singh Medical College, Jaipur, Rajasthan, India

## Abstract

**Background:**

Traumatic brain injury (TBI) is a major global health problem that disproportionally affects low- and middle-income countries. The needs for patients with TBI therefore may differ between levels of national development. We aimed to describe differences in epidemiology and acute care provision of TBI between India and Europe.

**Methods:**

We used data from two prospective observational registry studies – the Collaborative Indian NeuroTrauma Effectiveness Research in TBI (CINTER-TBI) and the Collaborative European NeuroTrauma Effectiveness Research in TBI (CENTER-TBI), which included TBI patients with an indication for brain CT-scan presenting to 65 centres across Europe and Israel and two trauma centres in India. We performed descriptive analyses of demographic, injury, and treatment characteristics and used random-effects logistic regression with covariate adjustment to examine the likelihood of acute neurosurgical interventions and in-hospital mortality.

**Results:**

We included 22 849 patients from CENTER-TBI and 3904 from CINTER-TBI. The median age in Europe was 55 years (IQR = 32–76) compared to 27 years (IQR = 18–40) in India. The most common cause of TBI in Europe were falls (n = 12150 (53%), while traffic incidents predominated in India (n = 2130 (55%)). The proportion of patients with severe TBI was higher in India (n = 867 (22%)) than in Europe (n = 1661 (7%). Professional pre-hospital care involving ambulance service was utilised by three-fourths (n = 17203 (75%)) of European and less than a one-tenth (n = 224 (6%)) of Indian patients in our sample. Patients with severe TBI were more likely to undergo surgical contusion/haematoma evacuation in India compared to Europe (OR = 2.0; 95% CI = 1.7–2.5) and Indian patients had higher odds of undergoing intracranial pressure monitor placement (OR = 2.3; 95% CI = 2.0–2.7). A primary decompressive craniectomy was likewise more often performed in the Indian cohort (OR = 5.1; 95% CI = 3.5–7.5). Discharge destinations in Europe included rehabilitation centres (n = 1261 (6%)) or nursing homes (n = 1208 (5%)), which was rarely the case in India (n = 13 (0%) and n = 9 (0%), respectively).

**Conclusions:**

Substantial disparities between India and Europe exist along the neurotrauma care chain, with both systems being likely to face unique features and challenges in the future.

Traumatic brain injury (TBI) is a major global health and socioeconomic problem affecting more than 50 million people each year and causing an estimated global financial burden of USD 400 billion [1]. Low-income and middle-income countries (LMICs) are disproportionately impacted, as about 90% of trauma-related deaths worldwide occur in such contexts [2]. In India, approximately 50% of trauma-related deaths are likely related to TBI [3,4], equalling one TBI-related death every three minutes. This growing public health crisis highlights a need for targeted resource allocation and regulations tailored to local needs and circumstances [1,5]. Therefore, the Indian Ministry of Health and Family Welfare produced an operational guideline in 2015 with the aim of reducing case-fatality rates from traffic incidents [6]. The most important challenge now, however, is the lack of availability of high-quality data on TBI epidemiology in India. Information systems in many places are manual and rudimentary, and no central trauma registries exists; in 2017 and 2022, the Lancet Neurology commissions on TBI emphasised the scarcity of up-to-date epidemiological data for patients with TBI, especially in LMICs, and particularly for individuals undergoing acute care interventions [1,5]. They advocated for improved characterisation of this population through extensive collaborative observational studies with harmonised data collection meant to allow for comparisons.

Recently, two globally coordinated research initiatives in the field of TBI have been established – the first being the prospective Collaborative Indian NeuroTrauma Effectiveness Research in TBI (CINTER-TBI) whose data collection was harmonised with the Collaborative European NeuroTrauma Effectiveness Research in TBI (CENTER-TBI) study. This harmonised approach has allowed comparisons of TBI care in India and Europe in large numbers. In our study, we aimed to describe differences in the epidemiology and acute care provision for TBI patients between India and Europe.

## METHODS

### Study population

**Figure 1:**
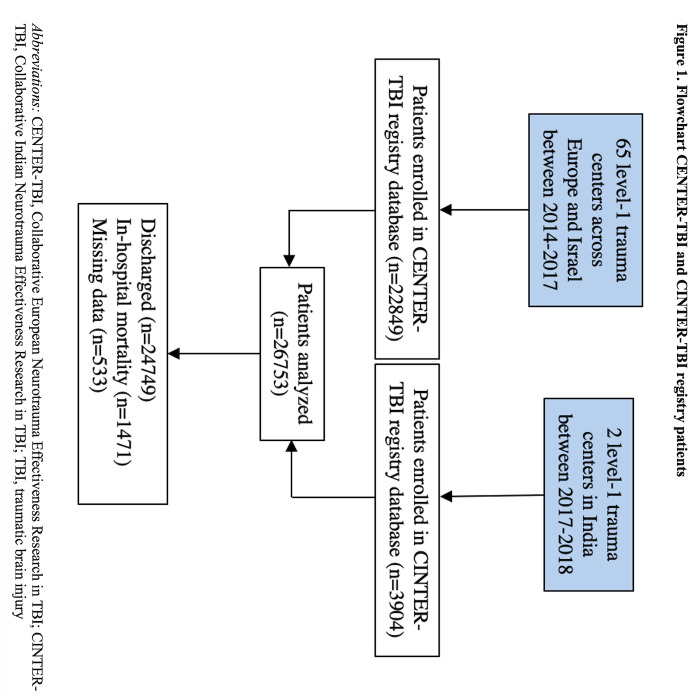
Flowchart CENTER-TBI and CINTER-TBI registry patients. CENTER-TBI – Collaborative European Neurotrauma Effectiveness Research in TBI, CINTER-TBI – Collaborative Indian Neurotrauma Effectiveness Research in TBI, TBI – traumatic brain injury.

The CENTER-TBI and CINTER-TBI registries were both prospective observational studies. The CENTER-TBI registry included patients of all ages with a clinical diagnosis of TBI presenting between 2014 and 2018 to one of 65 centres across Europe and Israel in whom computed tomography (CT) brain scan was being conducted [7,8]. There were otherwise no specific exclusion criteria. The CINTER-TBI registry, meanwhile, included patients meeting the same criteria who presented to one of two major governmental trauma centres in India, the All-India Institute of Medical Science in New Delhi and the Sawai Man Singh Hospital in Jaipur, from 2017 to 2018 (**Figure 1**). Both CENTER-TBI and CINTER-TBI were conducted in accordance with Good Clinical Practice standards (CPMP/ICH/135/95). All participating hospitals were level 1 trauma centres (Figure S1 in the **Online Supplementary Document**). Informed consent was not required for the use of the registry databases in our study, as only routinely collected clinical data were accessed with no patient identifiers being retained for analysis. Ethical approvals for the CENTER-TBI and CINTER-TBI studies were obtained from the medical ethics committees of all participating centres.

### Data collection

Local clinical research teams extracted patient registry variables from a patient’s clinical chart after they were admitted to the ward or intensive care unit (ICU) or were discharged. Data collection procedures were harmonised across studies: the same e-CRF was used in both studies, a common data dictionary was shared, and data were entered into identically structured databases. Extensive discussions between the principal investigators ensured a common understanding of data procedures and nomenclature, and study personnel received the same training provided during webinars, local site visits and through common manuals on standardised operating procedures. Data were entered into a web-based electronic case report form (Quesgen Systems Inc, Burlingame, USA) in an anonymised format, and were hosted on the International Neuroinformatics Coordinating Facility platform and extracted by a custom-made data management tool, Neurobot (RRID: SCR_01700) (INCF, Karolinska Institutet, Solna, Sweden). 

Here we used the datasets ‘CENTER Registry version 3.0’ and ‘CINTER India Registry’ which data on demographics (age and sex); pre-existing health status, including pre-injury anticoagulant and antiplatelet use; mechanism of injury; location of injury; severity of TBI and other traumatic injuries; CT brain findings; time from trauma to hospital arrival; time from trauma to CT scan; hospital or ICU length of stay; (timing of) emergency (neuro)surgical interventions; in-hospital mortality; and discharge destinations. Patients were included in one of three clinical care pathways after presentation and brain CT scan: home discharge from the emergency department or death, admission to the hospital but not to the ICU, or admission to the ICU.

### Statistical analysis

We summarised patient and injury characteristics using descriptive statistics, including medians (MDs) with interquartile ranges (IQRs) or numbers with percentages (rounded to nearest integer). Numerical data were compared with *t*-tests or Mann-Whitney U tests, depending on the normality of the distribution, while categorical data were analysed using χ^2^ tests. We approximated the likelihoods of undergoing intracranial surgery and in-hospital mortality with random-effects logistic regression models, incorporating the variables age, sex, baseline Glasgow Coma Score (GCS) and pupillary reactivity with a random-effect for (sub)continent to account for within-cluster correlations. We presented these findings using adjusted odds ratios (aORs) and their 95% confidence intervals (CI), with the corresponding ORs indicating the odds for surgery or mortality in India compared to Europe.

We considered *P*-values ≤0.05 as statistically significant. All analyses were performed in SPSS (version 28.0.1.0) or R (version 4.3.0). We reported our findings per the STROBE guidelines [9].

## RESULTS

### Demographics, injury mechanisms, and pre-hospital care

**Figure 2:**
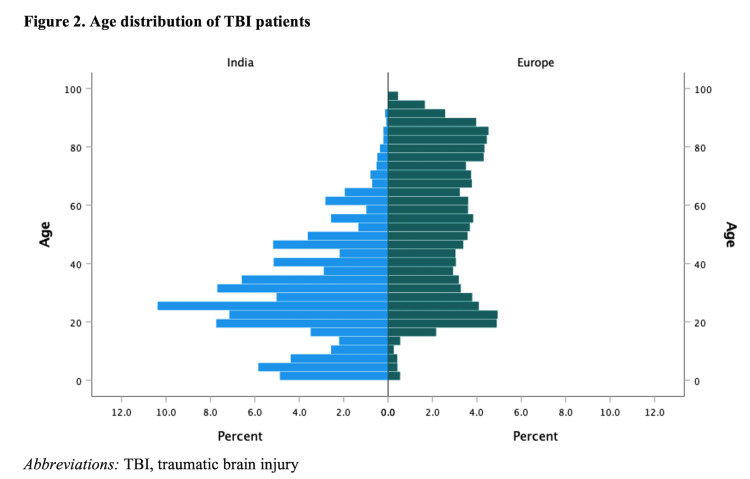
Age distribution of TBI patients. TBI – traumatic brain injury.

We included 22 849 patients from centres across Europe and Israel and 3904 from India. Substantial disparities were found in key demographic characteristics (**Table 1**). Median age was 55 (IQR = 32–76) years in Europe vs 27 (IQR = 18–40) years in India (**Figure 2**). The study population showed a higher male prevalence in India (n/N = 3010/3904 (77%) than in Europe (n/N = 13864/22849 (61%).

**Table 1:** Demographics and injury characteristics*

	Europe (n = 22849)	India (n = 3904)	*P*-value
**Age in years, MD (IQR)**	55 (32–76)	27 (18–40)	<0.001
**Age general population in years, MD (IQR)**	43 (37–50)	28 (22–35)	<0.001
**Age group**			<0.001
Paediatric (<18 y)	996 (4)	911 (23)	
Adult	13038 (57)	2804 (72)	
Older people (≥65 y)	8812 (39)	189 (5)	
Unknown/missing	3 (0)	0 (0)	
**Sex, n (%)**			<0.001
Male	13864 (61)	3010 (77)	
Female	8985 (39)	894 (23)	
**Pre-injury ASA classification**			<0.001
ASA I	9099 (40)	3733 (96)	
ASA II	6557 (29)	64 (2)	
ASA III	5347 (23)	88 (2)	
ASA IV	539 (2)	17 (0)	
Unknown/missing	1307 (6)	2 (0)	
**Anticoagulant use (incl. platelet inhibitors)**			<0.001
No	18068 (79)	3844 (99)	
Yes	2749 (12)	51 (1)	
Unknown/missing	2032 (9)	9 (0)	
**Injury mechanism**			<0.001
Road traffic incident	5913 (26)	2130 (55)	
Ground level fall	8639 (36)	210 (5)	
Fall from height (>1m/5 stairs)	3511 (15)	1231 (32)	
Assault/violence	2451 (11)	242 (6)	
Sport-related	784 (3)	12 (0)	
Other/unknown	1551 (7)	79 (2)	
**Location of accident**			<0.001
Home	8683 (38)	1334 (34)	
Street/traffic	7450 (33)	2228 (57)	
Public place	3555 (16)	270 (7)	
Sports field	724 (3)	3 (0)	
Other/unknown	2437 (11)	69 (2)	
**GCS at presentation**			<0.001
GCS 3–8	1661 (7)	867 (22)	
GCS 9–12	915 (4)	496 (13)	
GCS 13–15	18502 (81)	2521 (65)	
Unknown/missing	1771 (8)	20 (1)	
**Total ISS, MD (IQR)**	9 (4–17)	22 (9–25)	<0.001
**Head AIS**			<0.001
0	3264 (14)	8 (0)	
1–2	12038 (53)	300 (8)	
3	3754 (16)	2001 (51)	
4–5	3283 (14)	1587 (41)	
6	279 (1)	8 (0)	
Unknown/missing	231 (1)	0 (0)	
**Radiological findings on initial CT-scan**			<0.001
ASDH			
*No*	1950 (38)	1243 (66)	
*Small*	2126 (41)	492 (26)	
*Large*	1044 (20)	138 (7)	
*Unknown/missing*	58 (1)	2 (0)	
EDH			<0.001
*No*	4200 (81)	1430 (76)	
*Small*	645 (12)	297 (16)	
*Large*	254 (5)	147 (8)	
*Unknown/missing*	79 (1)	1 (0)	
Contusion			<0.001
*No*	1948 (38)	620 (33)	
*Small*	2539 (49)	1098 (59)	
*Large*	603 (12)	154 (8)	
*Unknown/missing*	88 (2)	3 (0)	
Traumatic SAH			<0.001
*No*	2468 (48)	1461 (78)	
*Yes*	2666 (51)	413 (22)	
*Unknown/missing*	44 (1)	1 (0)	
Midline shift			<0.001
*No*	3514 (68)	1348 (72)	
*0–4 mm*	580 (11)	277 (15)	
*≥5 mm*	918 (18)	250 (13)	
*Unknown/missing*	166 (3)	0 (0)	

AIS – Abbreviated Injury Scale, ASA – American Society of Anaesthesiologists classification system, ASDH – acute subdural haematoma, CT – computed tomography, EDH – extradural haematoma, GCS – Glasgow Coma Score, ICP – intracranial pressure, IQR – interquartile range, ISS – Injury Severity Score, MD – median, SAH – subarachnoid haemorrhage

*Values presented as n (%) unless specified otherwise.

Pre-injury American Society of Anaesthesiologists (ASA) Physical Status classification was higher in Europe compared to India for ASA 1 (n = 9099 (40%) vs n = 3733 (96%)), ASA II (n = 6557 (29%) vs n = 64 (2%)), ASA III (n = 5347 (23%) vs n = 88 (2%)) and ASA IV (n = 539 (2%) vs n = 17 (0%)). Antiplatelet or anticoagulant use was more common in European patients compared to the Indian patient population (n = 2749 (12%) vs n = 51 (1%)).

Road traffic injuries (n = 2130 (55%)) and falls from height (n = 1231 (32%)) were the most common mechanisms of injury in India, while ground level falls (n = 8639 (38%)), road traffic incidents (n = 5913 (26%)), and falls from height (n = 3511 (15%)) were most frequent in Europe. Falls from height were the most common injury mechanism among paediatric patients in India (n/N = 666/1029 (65%)) while falls from standing height were most common in older people in Europe (n/N = 5694/8812 (65%)) (Figure S2 in the **Online Supplementary Document**). With respect to the place of injury, there was a higher incidence of injuries sustained on the streets in India compared to Europe (2228 (57%) vs 7450 (33%)), alongside a comparable percentage of injuries at home (1334 (34%) vs 8683 (38%)). Subgroup analyses indicated that home-related injuries were mostly sustained by older adults in Europe (paediatric: n/N = 235/8683 (3%); adult: n/N = 2996/8683 (35%); older people: n/N = 5452/8683 (63%)), while in India, they were predominantly observed in paediatric patients (paediatric: n/N = 647/1334 (49%); adult: n/N = 598/1334 (45%); older people: n/N = 89/1334 (7%)). 

Pre-hospital professional care by ambulance service was provided to less than one-tenth of Indian patients (n/N = 223/3904 (6%)), compared to three-fourths of patients in Europe (n = 17203 (75%)) (**Table 2**). In Europe, helicopter assistance was additionally provided in 893 (4%) cases, while a mobile medical team was involved in 3800 (17%); these pre-hospital care types, meanwhile, were reported to be used in only one and two cases in India, respectively. In Europe, over three-fourths (n = 19 862 (87%)) of patients were transported directly from the scene of accident to the study centre, compared to just under two-thirds (n = 2423 (62%)) in India. Median time in hours from injury to the study hospital was shorter in Europe compared to India (MD = 1.6 (IQR = 0.8–5.7) vs 3.9 (MD = IQR = 1.6–9.8)). For patients with severe TBI, rates of pre-hospital intubation (n/N = 1062/1661 (64%) vs n/N = 48/867 (6%)) and ventilation (n/N = 967/1661 (58%) vs n/N = 39/867 (4%)) were higher in Europe compared to India.

**Table 2:** Acute care provision, outcomes, and process parameters*

	Europe (n = 22849)	India (n = 3904)	*P*-value
**Type of emergency care**			<0.001
No professional care	5259 (23)	3678 (94)	
Ambulance (without physician)	12510 (55)	223 (6)	
Helicopter service	893 (4)	1 (0)	
Mobile medical team	3800 (17)	2 (0)	
Not applicable	246 (1)	0 (0)	
Unknown/missing	141 (1)	0 (0)	
**Pre-hospital intubation in severe TBI patients**			<0.001
No	586 (35)	819 (95)	
Yes	1062 (64)	48 (6)	
Unknown/missing	13 (1)	0 (0)	
**Pre-hospital ventilation in severe TBI patients**			<0.001
No	659 (40)	828 (96)	
Yes	967 (58)	39 (4)	
Unknown/missing	35 (2)	0 (0)	
**Transport to study hospital**			<0.001
Primary referral	19862 (87)	2423 (62)	
Secondary referral from other hospital	2927 (13)	1481 (38)	
Unknown/missing	60 (0)	0 (0)	
**Time (hours) from injury to study hospital, MD (IQR)**	1.6 (0.8–5.7)	3.9 (1.6–9.8)	<0.001
**Time (hours) from injury to first CT-scan, MD (IQR)**	2.9 (1.7–7.0)	4.1 (2.3–10.0)	<0.001
**Care pathway**			<0.001
ED	9839 (43)	1258 (32)	
Admission	8571 (38)	1248 (32)	
ICU	4372 (19)	1398 (36)	
Unknown/missing	67 (0)	0 (0)	
**Key emergency intervention**†			<0.001
No	20029 (88)	2848 (73)	
Yes	2496 (11)	1056 (27)	
Unknown/missing	324 (1)	0 (0)	
**Intracranial surgery**			
Craniotomy for haematoma			<0.001
*No*	22019 (96)	3591 (92)	
*Yes*	830 (4)	313 (8)	
*Unknown/missing*	0 (0)	0 (0)	
Decompressive craniectomy			<0.001
*No*	22568 (99)	3515 (90)	
*Yes*	281 (1)	389 (10)	
*Unknown/missing*	0 (0)	0 (0)	
External ventricular drainage			<0.001
*No*	22691 (99)	3898 (100)	
*Yes*	158 (1)	6 (0)	
*Unknown/missing*	0 (0)	0 (0)	
ICP device insertion			<0.001
*No*	22063 (97)	3508 (90)	
*Yes*	786 (3)	396 (10)	
*Unknown/missing*	0 (0)	0 (0)	
**Time (hours) from SH presentation to ICP monitor placement, MD (IQR)**	3.1 (1.3–7.7)	8.6 (5.6–17)	<0.001
**Time (hours) from SH presentation to craniotomy, MD (IQR)**	2.2 (1.0–6.4)	4.3 (2.4–11)	<0.001
**Time (hours) from SH presentation to DC, MD (IQR)**	2.5 (1.1–12)	7.6 (4.0–18)	<0.001
**Time (hours) from SH presentation to EVD insertion, MD (IQR)**	5.7 (1.8–39)	4.7 (3.5–361)	0.83
**Time (hours) from CT-scan to ICP monitor placement, MD (IQR)**	4.3 (1.6–11)	7.1 (3.9–16)	<0.001
**Time (hours) from CT-scan to craniotomy, MD (IQR)**	3.6 (1.3–8.1)	5.0 (1.7–14)	0.007
**Time (hours) from CT-scan to DC, MD (IQR)**	3.6 (1.2–13)	6.5 (2.3–17)	0.0036
**Time (hours) from CT-scan to EVD insertion, MD (IQR)**	7.1 (1.7–35)	3.2 (1.6–360)	0.601
**Length (days) of hospital stay, MD (IQR)**	0.7 (0.2–5.5)	1.9 (0.2–7.6)	<0.001
**Length (days) of hospital stay for admitted patients, MD (IQR)**	4.0 (1.1–12)	2.5 (0.3–8.0)	<0.001
**Length (days) of ICU stay, MD (IQR)**	3.7 (1.3–12)	4.0 (1.8–7.3)	0.122
**Time (days) from injury to death, MD (IQR)**	3.4 (1.1–9.9)	4.6 (2.0–9.6)	0.001
**Time (days) from presentation at study hospital to death, MD (IQR)**	3.0 (0.9–9.2)	3.7 (1.7–8.6)	0.0087
**Time (days) from presentation to study hospital to death or discharge in severe TBI patients, MD (IQR)**	8.8 (1.8–24)	8.8 (3.5–18)	0.902
**Time (days) from presentation to study hospital to discharge in severe TBI patients, MD (IQR)**	16 (5.5–32)	13 (7.0–21)	0.009
**Status at discharge**			<0.001
Alive	21258 (93)	3491 (89)	
Dead	1058 (5)	413 (11)	
Unknown/missing	533 (2)	0 (0)	
**Discharge destination**			<0.001
Other hospital	2324 (10)	424 (11)	
Rehabilitation	1261 (6)	13 (0)	
Nursing home	1208 (5)	9 (0)	
Home	15962 (70)	3035 (78)	
Unknown/missing	2094 (9)	423 (11)	

ASDH – acute subdural haematoma, CT – computed tomography, DC – decompressive craniectomy, ED – emergency department, EDH – extradural haematoma, EVD – external ventricular drain, ICP – intracranial pressure, ICU – intensive care unit, IQR – interquartile range, MD – median, SH – study hospital, TBI – traumatic brain injury

*Values presented as n (%) unless specified otherwise.

†Defined as craniotomy for haematoma, DC, ICP device insertion, external ventricular drainage, damage control thoracotomy, damage control laparotomy, extraperitoneal pelvic packing, external fixation limb, interventional radiological procedure or other intervention

### Injury severity, radiological characteristics, and in-hospital care

**Figure 3:**
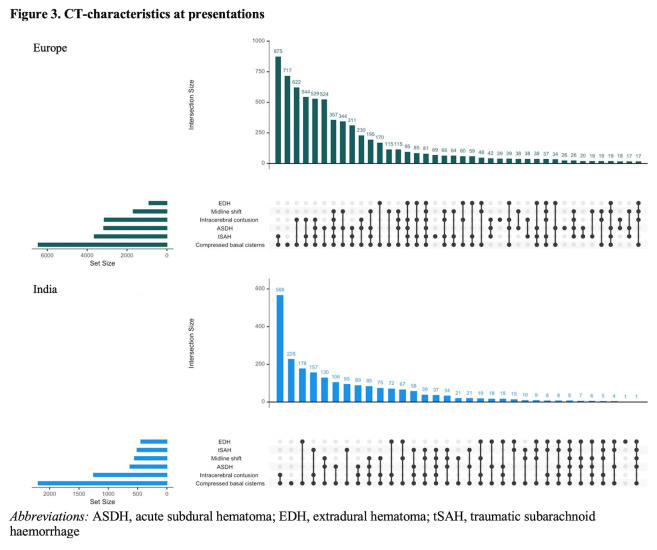
CT characteristics at presentations. ASDH – acute subdural haematoma, EDH – extradural haematoma, tSAH – traumatic subarachnoid haemorrhage.

Differences in severity of TBI, expressed by the GCS at presentation, were found between Europe and India for severe TBI (GCS = 3–8) (n = 1661 (7%) vs n = 867 (22%), moderate TBI (GCS 9–12) (n = 915 (4%) vs n = 496 (13%)) and mild TBI (GCS 13–15) (n = 18 502 (81%) vs n = 2521 (65%)) (**Table 1**; Figure S3 in the **Online Supplementary Document**). Total Injury Severity Score was higher in India (MD = 22, IQR = 9–25) compared to Europe (MD = 9 (IQR = 4–17)), as was the Head Abbreviated Injury Scale score (MD = 3 (IQR = 3–4) vs MD = 2 (IQR = 1–3)). Median time in hours from injury to the first CT-scan was shorter in Europe (MD = 2.9 (IQR = 1.7–7.0)) compared to India (MD = 4.1, IQR = 2.3–10.0)). Regarding patients with abnormalities on the first CT-scan, an acute subdural haematoma (n/N = 3170/5178 (61%) vs n/N = 630/1875 (34%)) and traumatic subarachnoid haemorrhage (n/N = 2666/5178 (51%) vs n/N = 413/1875 (22%)) were more common in Europe compared to India. Extradural haematoma, meanwhile, were more common in India (n/N = 444/1875 (24%)) compared to Europe (n/N = 899/5178 (17%)) (**Figure 3**).

An intracranial pressure (ICP) monitor was placed in 396 (10%) of patients in India versus 786 (3%) in Europe. Emergency intracranial surgery, defined as craniotomy for haematoma evacuation with or without primary decompressive craniectomy (DC), was performed in 695 (18%) of Indian patients versus 1049 (5%) European patients. After covariate adjustment, this translated into an increased likelihood of undergoing ICP monitor placement and emergency intracranial surgery in India (OR = 2.3; 95% CI = 2.0–2.7) compared to Europe (OR = 4.6; 95% CI = 4.0–5.2) (Table S1 in the **Online Supplementary Document**). The adjusted likelihood of external ventricular drain (EVD) insertion was significantly lower in India compared to Europe (n = 6 (0%) vs n = 158 (1%), OR = 0.1; 95% CI = 0.06–0.3). We found no significant difference for patients with severe TBI in India versus Europe regarding the likelihood of ICP monitor placement after confounding adjustment (n/N = 266/867 (31%) vs n/N = 399/1661 (24%), OR = 1.0; 95% CI = 0.8–1.2), while the probability of EVD insertion remained lower in India compared to Europe (n/N = 6/867 (1%) vs n/N = 93/1661 (6%), OR = 0.10; 95% CI = 0.05–0.2). Patients with severe TBI were more likely to undergo emergency intracranial surgery in India compared to Europe (n/N = 348/867 (40%) vs n/N = 396/1661 (24%), OR = 2.0; 1.7–2.5)), particularly when a large contusion was present (n/N = 45/80 (56%) vs n/N = 73/267 (27%), OR = 2.1; 95% CI = 1.2–3.8)). Severe TBI patients undergoing emergency intracranial surgery were more likely to receive a primary DC in India compared to Europe (n/N = 261/348 (75%) vs n/N = 150/396 (38%), OR = 5.1; 95% CI = 3.5–7.5), especially in cases with a large acute subdural haematoma (n/N = 37/85 (44%) vs n/N = 88/458 (19%), OR = 2.5; 95% CI = 1.2–5.0)).

Median time in hours from presentation at the study hospital to neurosurgical intervention was shorter in Europe compared to India for ICP monitor placement (MD = 3.1 (IQR = 1.3–7.7) vs MD = 8.6 (IQR = 5.6–17)), craniotomy for haematoma evacuation (MD = 2.2 (IQR = 1.0–6.4) vs MD = 4.3 (IQR = 2.4–11)) and DC (MD = 2.5 (IQR = 1.1–12) vs MD = 7.6 (IQR = 4.0–18)).

### Hospital stay, discharge status and post-hospital destinations

European patients were more often included in the emergency department care pathway (9839 (43%) vs 1258 (32%)) or admission pathway (8571 (38%) vs 1248 (32%)), while Indian patients were more often included in the ICU care pathway (1398 (36%) vs 4372 (19%)). For patients with severe TBI, median length of hospital stay was not significantly different between India and Europe (MD = 8.8 (IQR = 3.5–18) vs MD = 8.8 (IQR = 1.8–24)). The length of ICU stay in days was also comparable between Europe and India (MD = 3.7 (IQR = 1.3–12) vs MD = 4.0 (IQR = 1.8–7.3)) (**Table 2**). There were 413 (11%) cases of in-hospital mortality in India and 1058 (5%) in Europe. After multivariable adjustment, in-hospital mortality was still significantly lower in Europe compared to India (OR = 0.34; 95% CI = 0.28–0.40). This mortality difference was predominantly present for patients with severe TBI (n = 333 (38%) vs n = 504 (30%)) and less for moderate (n = 40 (8%) vs n = 68 (7%)) or mild (n = 37 (2%) vs n = 254 (1%)) TBI. The cause of death in Europe was more often the initial head injury (n/N = 580/1058 (55%)), while secondary intracranial damage was a more common cause in India (n/N = 195/413 (47%)) (Table S2 in the **Online Supplementary Document**). The median time in days from injury to death was significantly longer in India compared to Europe (MD = 4.6 (IQR = 2.0–9.6) vs MD = 3.4 (IQR = 1.1–9.9)), as was the time from presentation at the study hospital to death (MD = 3.7 (IQR = 1.7–8.6) vs MD = 3.0 (IQR = 0.9–9.2)). Similar percentages of patients in Europe (n = 2324 (10%)) and India (n = 424 (11%)) were discharged to other (referring) hospitals. In Europe compared to India, a larger proportion was discharged to either a rehabilitation centre (n = 1261 (6%) vs n = 13 (0%)) or nursing home (n = 1208 (5%) vs n = 9 (0%)). Just over two-thirds (n = 15 962 (70%() of European patients and over three-fourths (n = 3035 (78%)) of patients in India were discharged home (**Table 2**).

## DISCUSSION

CINTER-TBI and CENTER-TBI are large observational studies that describe the processes and structures of TBI care in India and Europe. In our analysis of their sample, we observed substantial disparities along the entire neurotrauma care chain spanning from injury to post-hospital care.

The significantly younger neurotrauma population, the corresponding lower ASA score, and the lower use of anticoagulants in India compared to Europe are in line with the current demographic pyramids. Furthermore, the age difference may be related to specific injury mechanisms. The increasingly older population experiencing falls from standing height is considered the main contributor to the TBI burden of disease in Europe [1,8,10]. This incidence is likely to increase even further, as the share of older people in the European Union is expected to double to 30% by 2060 [11].

A similar demographic trend, although to a lesser extent and occurring much later, is expected in India due to future ageing of the population [12]. In our study, however, we observed a high incidence of paediatric TBI in India, for which the main cause was falls from height. They have been mentioned as the leading cause of death among young children in India ever since the decline in infection-related mortality [13]. These data have inspired the All-India Institute of Medical Science to launch the ‘Safe Balcony, Safe Child’ campaign with the aim to increase awareness and offer specific tools to prevent TBI in young children and thereby reduce the burden of disease for this specific patient group [14].

Road traffic incidents remain the leading cause of TBI among adults in India. This follows previous estimations that approximately 60% of head injuries in India are due to traffic incidents [15]. As seen in fast-growing economies, this can be attributed to a rapid rise in motorised vehicles on the road without analogous development in infrastructure, precautions, and legislation [16]. In Europe, the number of road traffic fatalities been decreasing for over 20 years, mainly due to coordinated governmental regulations [17]. In India, various coordinated public campaigns by organisations such as the Neurotrauma Society of India and the Neurological Society of India, together with non-governmental organisations, have aimed to address these issues by focussing on key subjects like road- and vehicle safety, public awareness, and helmet use [18]. These initiatives have yielded tangible results such as helmet laws and ‘Good Samaritan Laws’ protecting civilians who aid road traffic injury victims from financial or legal prosecution. Despite the ever-increasing population and vehicle numbers, the plateaued incidence of road traffic injury related deaths in India around 2010, is perhaps partly due to these initiatives [18].

Besides varying patient populations and trauma mechanisms, we found key differences in regards to the pre-hospital phase. Ambulance services and mobile medical teams were generally available in Europe, while professional pre-hospital care was present in only 6% of cases in India. Even though high-level ambulance services, often financed by local governmental or private institutions, are increasingly being developed throughout India, many rural and semi-urban areas still lack such facilities [19,20]. The current data show that most European patients were transported directly to a level 1 trauma centre, while patients in India more often presented to a local hospital first. Moreover, time from injury to presentation at the study hospital (a level 1 trauma centre) and time to the first CT-scan were found to be significantly longer in India. This disparity can probably be attributed to the fact that many rural areas in India are located several hundreds of kilometres from a designated hospital with neurotrauma care facilities. It has been reported that up to one-third of all general trauma patients in India presents to a hospital more than 24 hours after trauma [21]. Improving timely access to complex neurotrauma care by advancing the pre-hospital medical infrastructure with the aim to make the ‘golden hour’ concept for trauma care within reach will continue to be a priority for policy makers in India and will likely improve functional outcomes and quality of life of neurotrauma patients [22].

Indian patients were more severely injured than European patients upon presentation to a designated neurotrauma centre. It is likely that Indian patients sustaining mild TBI experience a higher threshold due to geographical, financial, or other practical factors to seek specialised care than their European counterparts. This could bias the data towards more severe TBI in India [23]. Also, long-distance transports to level 1 neurotrauma centres could have exacerbated the patients’ condition at hospital presentation. Conversely, patients with severe TBI may not survive such long-distance transports, which could potentially lead to selection bias in the opposite direction.

The first CT scan after arrival at the study hospital more often showed an epidural haematoma in Indian patients compared to an acute subdural haematoma in European patients. This probably reflects differences in population age and trauma mechanisms, with the corresponding susceptibility to different types of intracranial injuries (i.e., younger patients after high-energetic trauma vs older patients after low-velocity falls, respectively). After adjusting for baseline variables and injury severity, patients seemed more likely to undergo emergency intracranial surgery in India compared to Europe, especially when a large intracranial contusion was present. Severe TBI patients undergoing intracranial surgery were more likely to undergo primary DC in India vs Europe. This could reflect a cultural difference in aggressiveness of emergency neurosurgical care for these patients between India and Europe, although residual (unmeasured) confounding might also be an explanation [20,24–26]. The likelihood of ICP monitor placement was higher in India compared to Europe, while the reverse was true for EVD insertion. This could be partly explained by between-centre treatment preferences regarding the use of intraparenchymal pressure monitors vs EVDs for monitoring ICP [27,28]. Furthermore, local monitoring facilities and resources may have influenced neurosurgical decision-making for TBI patients, although no firm conclusions regarding this can be drawn from the current data.

Time from arrival at the study hospital and from the first CT scan to ICP monitor placement, craniotomy, or DC was significantly longer in India compared to Europe. This potential reason for this could be differences in trauma and hospital logistics. The large-scale level 1 trauma centres in India with their corresponding geographical drainage areas may serve a much larger population than European hospitals. For patients with severe TBI, total length-of-stay in the level 1 trauma hospital or in the ICU were not significantly different between Europe and India, despite the greater severity of TBI in India and the larger proportion of Indian patients being included in the ICU care pathway. This could reflect disparities in medical resource availability, forcing similarly injured patients in India to be discharged earlier than in Europe. However, additional data are required to further explore this hypothesis.

We also observed large differences in discharge destinations and post-hospital care. While there was availability of rehabilitation centres and nursing homes in Europe, patients in India were hardly referred to such facilities and were either transferred to another (referring) hospital or discharged home. Rehabilitation is still a scarce commodity in India, as shortages across the care chain and lack of resources often leave no other option than home discharge [22,29]. This issue was also addressed during the World Health Organization (WHO) meeting ‘Rehabilitation 2030: a call for action’, which focussed on the unmet rehabilitation needs around the world, with a particular emphasis on LMICs [30]. Simultaneously, European healthcare systems including post-hospital care facilities are also facing a crisis. Increasing shortages of healthcare staff together with the rapid increase in older people requiring neurotrauma care has been described as ‘a ticking time bomb’ by the WHO, and some have even forecasted the impending ‘collapse of the European healthcare system’ if prompt action is not taken [31,32].

The strengths of this study are the large patient cohorts and the harmonised data collection procedures, which ensured data consistency and facilitated comparisons across diverse geographical locations. Moreover, the coverage of multiple facets of TBI care, including demographics, injury mechanisms, pre-hospital care, in-hospital treatments, and discharge destinations, have not been reported in prior studies. By directly comparing TBI care chains between India and Europe, this study addresses an evident gap in current literature.

Our study also has several limitations. First, all participating hospitals were level 1 neurotrauma centres, mostly located in urban areas, with access to state-of-the-art neuroimaging, ICUs, and neurosurgical facilities. Hence, our results may not be generalisable to TBI patients presenting to hospitals in more rural settings, where access to specialised neurotrauma care is virtually absent. Second, our findings should be interpreted with consideration of the different demographic pyramids between India and Europe. Third, several biases may be present. Selection bias caused by unmeasured differences in patient populations and injury severity remains possible. Confounding may explain differences in likelihood of the interventions, despite statistical adjustment. Finally, functional outcomes were not available.

## CONCLUSIONS

We found substantial disparities between India and Europe across the entire chain of neurotrauma care. However, both healthcare systems have unique features and will likely experience their own challenges in the future; their thorough comparison could therefore provide a reliable evidence base for their improvement. While India may take further steps towards centrally-coordinated, timely neurotrauma care for all, European countries will have to accommodate the rapidly ageing population. To combat these challenges, centralised governmental legislation and financial support should be tailored to local needs in Europe and India, which should be reassessed by continuous monitoring with targeted high-quality data analogous to CENTER-TBI and CINTER-TBI. Our findings may inform legislative discussions, stimulate initiatives combating the ever-increasing public health burden of TBI, and provide a reliable foundation for further collaborative Indian-European initiatives.

## Additional material


Online Supplementary Document
