# Supplementary material for: Disparities in casemix, acute interventions, discharge destinations and mortality of patients with traumatic brain injury between Europe and India
Source: J Glob Health. 2024 Nov 11;14:04227. doi: 10.7189/jogh.14.04227 (PMC11614187; doi:10.7189/jogh.14.04227)
Supplement: Online Supplementary Document [file jogh-14-04227-s001.pdf]

Online Supplementary Document

Table S1. Likelihood of emergency neurosurgical care in India vs Europe

	Unadjusted odds ratio (OR) (95%CI)	Adjusted odds ratio (OR)[b] (95%CI)
Likelihood of undergoing intracranial surgery in India versus Europe	4.3 (3.9-4.7)	4.3 (3.8-4.9)
ICP monitor placement	3.2 (2.8-3.6)	2.3 (2.0-2.7)
Emergency intracranial surgery[a]	4.5 (4.1-5.0)	4.6 (4.0-5.2)
Primary DC vs craniotomy	3.5 (2.8-4.3)	4.4 (3.4-5.9)
EVD insertion	0.2 (0.1-0.5)	0.1 (0.06-0.3)
Likelihood of undergoing cranial surgery in India versus Europe for severe TBI	2.0 (1.7-2.4)	1.7 (1.4-2.0)
ICP monitor placement	1.4 (1.2-1.7)	1.0 (0.8-1.2)
Emergency intracranial surgery[a]	2.1 (1.8-2.6)	2.0 (1.7-2.5)
Emergency intracranial surgery for ASDH	2.0 (1.2-3.3)	1.1 (0.6-1.9)
Emergency intracranial surgery for EDH	1.7 (0.8-3.7)	0.8 (0.3-2.0)
Emergency intracranial surgery for contusions	3.4 (2.0-5.7)	2.1 (1.2-3.8)
Primary DC vs craniotomy	4.9 (3.6-6.8)	5.1 (3.5-7.5)
Primary DC vs craniotomy in ASDH surgery	3.0 (1.6-5.5)	2.5 (1.2-5.0)
Primary DC vs craniotomy in EDH surgery	1.4 (0.6-3.4)	2.4 (0.8-7.3)
Primary DC vs craniotomy in contusion surgery	1.7 (0.8-3.8)	2.3 (0.9-5.5)
EVD insertion	0.1 (0.05-0.3)	0.1 (0.05-0.2)

[a] Defined as craniotomy for hematoma evacuation and/or decompressive craniectomy
[b] The odds ratio was adjusted for age, sex, baseline GCS and pupillary reactivity
Abbreviations: ASDH, acute subdural hematoma; CI, confidence interval; DC, decompressive craniectomy; EDH, extradural hematoma; EVD, external ventricular drain; GCS, Glasgow Coma Score; ICP, intracranial pressure; OR, odds ratio; TBI, traumatic brain injury

Table S2. Causes of in-hospital mortality

	Europe (n=1058)	India (n=413)	P value
Initial head injury, n (%)	580 (55)	142 (34)	<0.001
Secondary intracranial damage, n (%)	141 (13)	195 (47)	
Systemic trauma, n (%)	72 (7)	14 (3)	
Medical complications, n (%)	146 (14)	14 (3)	
Unknown/other, n (%)	119 (11)	48 (12)	

Figure S1. Participating level-1 trauma centers in Europe (A) and India (B)

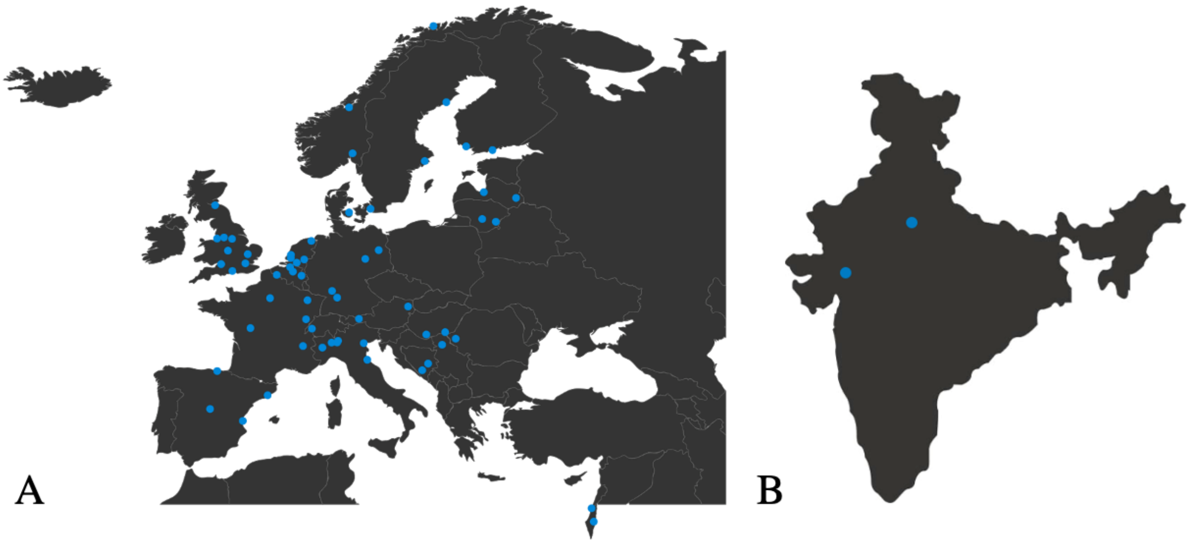

Figure S2. Injury mechanism per age group

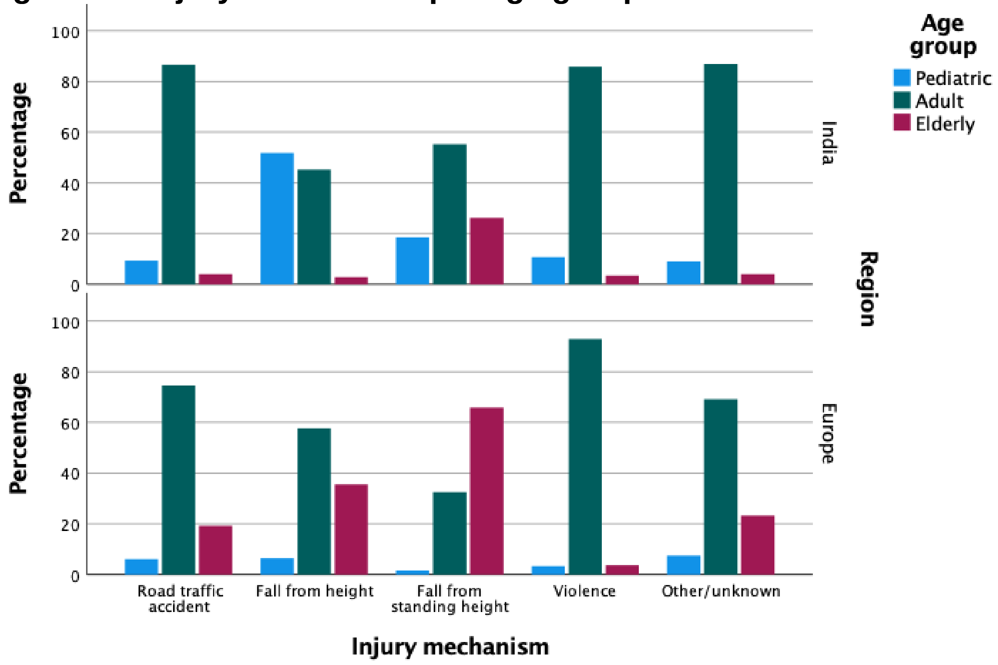

Figure S3. TBI severity at presentation

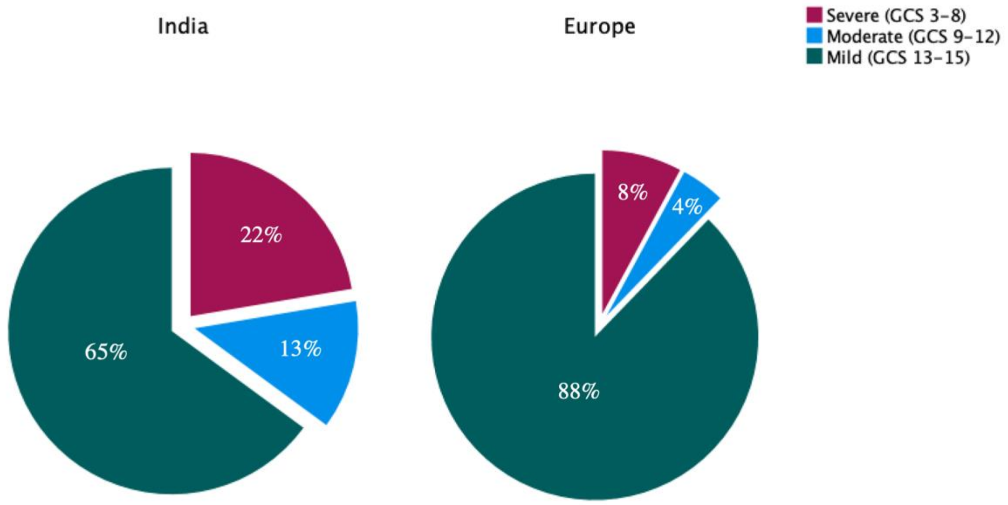

Abbreviations: GCS, Glasgow Coma Score; TBI, traumatic brain injury

Supplemental material S1. CENTER-TBI and CINTER-TBI participants and investigators

The CENTER-TBI participants and investigators:

Cecilia Åkerlund[1], Krisztina Amrein[2], Nada Andelic[3], Lasse Andreassen[4], Audny Anke[5], Anna Antoni[6], Gérard Audibert[7], Philippe Azouvi[8], Maria Luisa Azzolini[9], Ronald Bartels[10], Pál Barzó[11], Romuald Beauvais[12], Ronny Beer[13], Bo-Michael Bellander[14], Antonio Belli[15], Habib Benali[16], Maurizio Berardino[17], Luigi Beretta[9], Morten Blaabjerg[18], Peter Bragge[19], Alexandra Brazinova[20], Vibeke Brinck[21], Joanne Brooker[22], Camilla Brorsson[23], Andras Buki[24], Monika Bullinger[25], Manuel Cabeleira[26], Alessio Caccioppola[27], Emiliana Calappi [27], Maria Rosa Calvi[9], Peter Cameron[28], Guillermo Carbayo Lozano[29], Marco Carbonara[27], Simona Cavallo[17], Giorgio Chevallard[30], Arturo Chieregato[30], Giuseppe Citerio[31, 32], Hans Clusmann[33], Mark Coburn[34], Jonathan Coles[35], Jamie D. Cooper[36], Marta Correia[37], Amra Čović [38], Nicola Curry[39], Endre Czeiter[24], Marek Czosnyka[26], Claire Dahyot-Fizelier[40], Paul Dark[41], Helen Dawes[42], Véronique De Keyser[43], Vincent Degos[16], Francesco Della Corte[44], Hugo den Boogert[10], Bart Depreitere[45], Đula Đilvesi [46], Abhishek Dixit[47], Emma Donoghue[22], Jens Dreier[48], Guy-Loup Dulière[49], Ari Ercole[47], Patrick Esser[42], Erzsébet Ezer[50], Martin Fabricius[51], Valery L. Feigin[52], Kelly Foks[53], Shirin Frisvold[54], Alex Furmanov[55], Pablo Gagliardo[56], Damien Galanaud[16], Dashiell Gantner[28], Guoyi Gao[57], Pradeep George[58], Alexandre Ghuysen[59], Lelde Giga[60], Ben Glocker[61], Jagoš Golubovic[46], Pedro A. Gomez [62], Johannes Gratz[63], Benjamin Gravesteijn[64], Francesca Grossi[44], Russell L. Gruen[65], Deepak Gupta[66], Juanita A. Haagsma[64], Iain Haitsma[67], Raimund Helbok[13], Eirik Helseth[68], Lindsay Horton [69], Jilske Huijben[64], Peter J. Hutchinson[70], Bram Jacobs[71], Stefan Jankowski[72], Mike Jarrett[21], Ji-yao Jiang[58], Faye Johnson[73], Kelly Jones[52], Mladen Karan[46], Angelos G. Kolias[70], Erwin Kompanje[74], Daniel Kondziella[51], Evgenios Kornaropoulos[47], Lars-Owe Koskinen[75], Noémi Kovács[76], Ana Kowark[77], Alfonso Lagares[62], Linda Lanyon[58], Steven Laureys[78], Fiona Lecky[79, 80], Didier Ledoux[78], Rolf Lefering[81], Valerie Legrand[82], Aurelie Lejeune[83], Leon Levi[84], Roger Lightfoot[85], Hester Lingsma[64], Andrew I.R. Maas[43,86], Ana M. Castaño-León[62], Marc Maegele[87], Marek Majdan[20], Alex Manara[88], Geoffrey Manley[89], Costanza Martino[90], Hugues Maréchal[49], Julia Mattern[91], Catherine McMahon[92], Béla Melegh[93], David Menon[47], Tomas Menovsky[43,86], Ana Mikolic[64], Benoit Misset[78], Visakh Muraleedharan[58], Lynnette Murray[28], Ancuta Negru[94], David Nelson[1], Virginia Newcombe[47], Daan Nieboer[64], József Nyirádi[2], Otesile Olubukola[79], Matej Oresic[95], Fabrizio Ortolano[27], Aarno Palotie[96, 97, 98], Paul M. Parizel[99], Jean-François Payen[100], Natascha Perera[12], Vincent Perlbarg[16], Paolo Persona[101], Wilco Peul[102], Anna Piippo-Karjalainen[103], Matti Pirinen[96], Dana Pisica[64], Horia Ples[94], Suzanne Polinder[64], Inigo Pomposo[29], Jussi P. Posti [104], Louis Puybasset[105], Andreea Radoi [106], Arminas Ragauskas[107], Rahul Raj[103], Malinka Rambadagalla[108], Isabel Retel Helmrich[64], Jonathan Rhodes[109], Sylvia Richardson[110], Sophie Richter[47], Samuli Ripatti[96], Saulius Rocka[107], Cecilie Roe[111], Olav Roise[112,113], Jonathan Rosand[114], Jeffrey V. Rosenfeld[115], Christina Rosenlund[116], Guy Rosenthal[55], Rolf Rossaint[77], Sandra Rossi[101], Daniel Rueckert[61] Martin Rusnák[117], Juan Sahuquillo[106], Oliver Sakowitz[91, 118], Renan Sanchez-Porras[118], Janos Sandor[119], Nadine Schäfer[81],

Silke Schmidt[120], Herbert Schoechl[121], Guus Schoonman[122], Rico Frederik Schou[123], Elisabeth Schwendenwein[6], Charlie Sewalt[64], Ranjit D. Singh[102], Toril Skandsen[124, 125], Peter Smielewski[26], Abayomi Sorinola[126], Emmanuel Stamatakis[47], Simon Stanworth[39], Robert Stevens[127], William Stewart[128], Ewout W. Steyerberg[64, 129], Nino Stocchetti[130], Nina Sundström[131], Riikka Takala[132], Viktória Tamás[126], Tomas Tamosuitis[133], Mark Steven Taylor[20], Aurore Thibaut[78], Braden Te Ao[52], Olli Tenovuo[104], Alice Theadom[52], Matt Thomas[88], Dick Tibboel[134], Marjolein Timmers[74], Christos Tolias[135], Tony Trapani[28], Cristina Maria Tudora[94], Andreas Unterberg[91], Peter Vajkoczy[136], Shirley Vallance[28], Egils Valeinis[60], Zoltán Vámos[50], Mathieu van der Jagt[137], Gregory Van der Steen[43], Joukje van der Naalt[71], Jeroen T.J.M. van Dijck[102], Inge A. M. van Erp[102], Thomas A. van Essen[102], Wim Van Hecke[138], Caroline van Heugten[139], Ernest van Veen[64], Thijs Vande Vyvere[140], Roel P. J. van Wijk[102], Alessia Vargiolu[32], Emmanuel Vega[83], Kimberley Velt[64], Jan Verheyden[138], Paul M. Vespa[141], Anne Vik[124, 142], Rimantas Vilcinis[133], Victor Volovici[67], Nicole von Steinbüchel[38], Daphne Voormolen[64], Petar Vulekovic[46], Kevin K.W. Wang[143], Daniel Whitehouse[47], Eveline Wiegers[64], Guy Williams[47], Lindsay Wilson[69], Stefan Winzeck[47], Stefan Wolf[144], Zhihui Yang[114], Peter Ylén[145], Alexander Younsi[91], Frederick A. Zeiler[47,146], Veronika Zelinkova[20], Agate Ziverte[60], Tommaso Zoerle[27]

[1] Department of Physiology and Pharmacology, Section of Perioperative Medicine and Intensive Care, Karolinska Institutet, Stockholm, Sweden
[2] János Szentágothai Research Centre, University of Pécs, Pécs, Hungary
[3] Division of Clinical Neuroscience, Department of Physical Medicine and Rehabilitation, Oslo University Hospital and University of Oslo, Oslo, Norway
[4] Department of Neurosurgery, University Hospital Northern Norway, Tromso, Norway
[5] Department of Physical Medicine and Rehabilitation, University Hospital Northern Norway, Tromso, Norway
[6] Trauma Surgery, Medical University Vienna, Vienna, Austria
[7] Department of Anesthesiology & Intensive Care, University Hospital Nancy, Nancy, France
[8] Raymond Poincare hospital, Assistance Publique – Hopitaux de Paris, Paris, France
[9] Department of Anesthesiology & Intensive Care, S Raffaele University Hospital, Milan, Italy
[10] Department of Neurosurgery, Radboud University Medical Center, Nijmegen, The Netherlands
[11] Department of Neurosurgery, University of Szeged, Szeged, Hungary
[12] International Projects Management, ARTTIC, Munchen, Germany
[13] Department of Neurology, Neurological Intensive Care Unit, Medical University of Innsbruck, Innsbruck, Austria
[14] Department of Neurosurgery & Anesthesia & intensive care medicine, Karolinska University Hospital, Stockholm, Sweden
[15] NIHR Surgical Reconstruction and Microbiology Research Centre, Birmingham, UK
[16] Anesthesie-Réanimation, Assistance Publique – Hopitaux de Paris, Paris, France
[17] Department of Anesthesia & ICU, AOU Città della Salute e della Scienza di Torino - Orthopedic and Trauma Center, Torino, Italy
[18] Department of Neurology, Odense University Hospital, Odense, Denmark
[19] BehaviourWorks Australia, Monash Sustainability Institute, Monash University, Victoria, Australia
[20] Department of Public Health, Faculty of Health Sciences and Social Work, Trnava University, Trnava, Slovakia
[21] Quesgen Systems Inc., Burlingame, California, USA
[22] Australian & New Zealand Intensive Care Research Centre, Department of Epidemiology and Preventive Medicine, School of Public Health and Preventive Medicine, Monash University, Melbourne, Australia
[23] Department of Surgery and Perioperative Science, Umeå University, Umeå, Sweden
[24] Department of Neurosurgery, Medical School, University of Pécs, Hungary and Neurotrauma Research Group, János Szentágothai Research Centre, University of Pécs, Hungary
[25] Department of Medical Psychology, Universitätsklinikum Hamburg-Eppendorf, Hamburg, Germany
[26] Brain Physics Lab, Division of Neurosurgery, Dept of Clinical Neurosciences, University of Cambridge, Addenbrooke's Hospital, Cambridge, UK
[27] Neuro ICU, Fondazione IRCCS Cà Granda Ospedale Maggiore Policlinico, Milan, Italy
[28] ANZIC Research Centre, Monash University, Department of Epidemiology and Preventive Medicine, Melbourne, Victoria, Australia
[29] Department of Neurosurgery, Hospital of Cruces, Bilbao, Spain
[30] NeuroIntensive Care, Niguarda Hospital, Milan, Italy
[31] School of Medicine and Surgery, Università Milano Bicocca, Milano, Italy

[32] NeuroIntensive Care Unit, Department Neuroscience, IRCCS Fondazione San Gerardo dei Tintori, Monza, Italy
[33]Department of Neurosurgery, Medical Faculty RWTH Aachen University, Aachen, Germany
[34] Department of Anesthesiology and Intensive Care Medicine, University Hospital Bonn, Bonn, Germany
[35] Department of Anesthesia & Neurointensive Care, Cambridge University Hospital NHS Foundation Trust, Cambridge, UK
[36] School of Public Health & PM, Monash University and The Alfred Hospital, Melbourne, Victoria, Australia
[37] Radiology/MRI department, MRC Cognition and Brain Sciences Unit, Cambridge, UK
[38] Institute of Medical Psychology and Medical Sociology, Universitätsmedizin Göttingen, Göttingen, Germany
[39] Oxford University Hospitals NHS Trust, Oxford, UK
[40] Intensive Care Unit, CHU Poitiers, Potiers, France
[41] University of Manchester NIHR Biomedical Research Centre, Critical Care Directorate, Salford Royal Hospital NHS Foundation Trust, Salford, UK
[42] Movement Science Group, Faculty of Health and Life Sciences, Oxford Brookes University, Oxford, UK
[43] Department of Neurosurgery, Antwerp University Hospital, Edegem, Belgium
[44] Department of Anesthesia & Intensive Care, Maggiore Della Carità Hospital, Novara, Italy
[45] Department of Neurosurgery, University Hospitals Leuven, Leuven, Belgium
[46] Department of Neurosurgery, Clinical centre of Vojvodina, Faculty of Medicine, University of Novi Sad, Novi Sad, Serbia
[47] Division of Anaesthesia, University of Cambridge, Addenbrooke's Hospital, Cambridge, UK
[48] Center for Stroke Research Berlin, Charité – Universitätsmedizin Berlin, corporate member of Freie Universität Berlin, Humboldt-Universität zu Berlin, and Berlin Institute of Health, Berlin, Germany
[49] Intensive Care Unit, CHR Citadelle, Liège, Belgium
[50] Department of Anaesthesiology and Intensive Therapy, University of Pécs, Pécs, Hungary
[51] Departments of Neurology, Clinical Neurophysiology and Neuroanesthesiology, Region Hovedstaden Rigshospitalet, Copenhagen, Denmark
[52] National Institute for Stroke and Applied Neurosciences, Faculty of Health and Environmental Studies, Auckland University of Technology, Auckland, New Zealand
[53] Department of Neurology, Erasmus MC, Rotterdam, the Netherlands
[54] Department of Anesthesiology and Intensive care, University Hospital Northern Norway, Tromso, Norway
[55] Department of Neurosurgery, Hadassah-hebrew University Medical center, Jerusalem, Israel
[56] Fundación Instituto Valenciano de Neurorrehabilitación (FIVAN), Valencia, Spain
[57] Department of Neurosurgery, Shanghai Renji hospital, Shanghai Jiaotong University/school of medicine, Shanghai, China
[58] Karolinska Institutet, INCF International Neuroinformatics Coordinating Facility, Stockholm, Sweden
[59] Emergency Department, CHU, Liège, Belgium
[60] Neurosurgery clinic, Pauls Stradins Clinical University Hospital, Riga, Latvia
[61] Department of Computing, Imperial College London, London, UK
[62] Department of Neurosurgery, Hospital Universitario 12 de Octubre, Madrid, Spain
[63] Department of Anesthesia, Critical Care and Pain Medicine, Medical University of Vienna, Austria
[64] Department of Public Health, Erasmus Medical Center-University Medical Center, Rotterdam, The Netherlands
[65] College of Health and Medicine, Australian National University, Canberra, Australia
[66] Department of Neurosurgery, Neurosciences Centre & JPN Apex trauma centre, All India Institute of Medical Sciences, New Delhi-110029, India
[67] Department of Neurosurgery, Erasmus MC, Rotterdam, the Netherlands
[68] Department of Neurosurgery, Oslo University Hospital, Oslo, Norway
[69] Division of Psychology, University of Stirling, Stirling, UK
[70] Division of Neurosurgery, Department of Clinical Neurosciences, Addenbrooke's Hospital & University of Cambridge, Cambridge, UK
[71] Department of Neurology, University of Groningen, University Medical Center Groningen, Groningen, Netherlands
[72] Neurointensive Care , Sheffield Teaching Hospitals NHS Foundation Trust, Sheffield, UK
[73] Salford Royal Hospital NHS Foundation Trust Acute Research Delivery Team, Salford, UK

[74] Department of Intensive Care and Department of Ethics and Philosophy of Medicine, Erasmus Medical Center, Rotterdam, The Netherlands
[75] Department of Clinical Neuroscience, Neurosurgery, Umeå University, Umeå, Sweden
[76] Hungarian Brain Research Program - Grant No. KTIA_13_NAP-A-II/8, University of Pécs, Pécs, Hungary
[77] Department of Anaesthesiology, University Hospital of Aachen, Aachen, Germany
[78] Cyclotron Research Center , University of Liège, Liège, Belgium
[79] Centre for Urgent and Emergency Care Research (CURE), Health Services Research Section, School of Health and Related Research (ScHARR), University of Sheffield, Sheffield, UK
[80] Emergency Department, Salford Royal Hospital, Salford UK
[81] Institute of Research in Operative Medicine (IFOM), Witten/Herdecke University, Cologne, Germany
[82] VP Global Project Management CNS, ICON, Paris, France
[83] Department of Anesthesiology-Intensive Care, Lille University Hospital, Lille, France
[84] Department of Neurosurgery, Rambam Medical Center, Haifa, Israel
[85] Department of Anesthesiology & Intensive Care, University Hospitals Southhampton NHS Trust, Southhampton, UK
[86] Department of Translational Neuroscience, Faculty of Medicine and Health Science, University of Antwerp, Antwerp, Belgium
[87] Cologne-Merheim Medical Center (CMMC), Department of Traumatology, Orthopedic Surgery and Sportmedicine, Witten/Herdecke University, Cologne, Germany
[88] Intensive Care Unit, Southmead Hospital, Bristol, Bristol, UK
[89] Department of Neurological Surgery, University of California, San Francisco, California, USA
[90] Department of Anesthesia & Intensive Care,M. Bufalini Hospital, Cesena, Italy
[91] Department of Neurosurgery, University Hospital Heidelberg, Heidelberg, Germany
[92] Department of Neurosurgery, The Walton centre NHS Foundation Trust, Liverpool, UK
[93] Department of Medical Genetics, University of Pécs, Pécs, Hungary
[94] Department of Neurosurgery, Emergency County Hospital Timisoara , Timisoara, Romania
[95] School of Medical Sciences, Örebro University, Örebro, Sweden
[96] Institute for Molecular Medicine Finland, University of Helsinki, Helsinki, Finland
[97] Analytic and Translational Genetics Unit, Department of Medicine; Psychiatric & Neurodevelopmental Genetics Unit, Department of Psychiatry; Department of Neurology, Massachusetts General Hospital, Boston, MA, USA
[98] Program in Medical and Population Genetics; The Stanley Center for Psychiatric Research, The Broad Institute of MIT and Harvard, Cambridge, MA, USA
[99] Department of Radiology, University of Antwerp, Edegem, Belgium
[100] Department of Anesthesiology & Intensive Care, University Hospital of Grenoble, Grenoble, France
[101] Department of Anesthesia & Intensive Care, Azienda Ospedaliera Università di Padova, Padova, Italy
[102] Dept. of Neurosurgery, Leiden University Medical Center, Leiden, The Netherlands and Dept. of Neurosurgery, Medical Center Haaglanden, The Hague, The Netherlands
[103] Department of Neurosurgery, Helsinki University Central Hospital
[104] Division of Clinical Neurosciences, Department of Neurosurgery and Turku Brain Injury Centre, Turku University Hospital and University of Turku, Turku, Finland
[105] Department of Anesthesiology and Critical Care, Pitié -Salpêtrière Teaching Hospital, Assistance Publique, Hôpitaux de Paris and University Pierre et Marie Curie, Paris, France
[106] Neurotraumatology and Neurosurgery Research Unit (UNINN), Vall d'Hebron Research Institute, Barcelona, Spain
[107] Department of Neurosurgery, Kaunas University of technology and Vilnius University, Vilnius, Lithuania
[108] Department of Neurosurgery, Rezekne Hospital, Latvia
[109] Department of Anaesthesia, Critical Care & Pain Medicine NHS Lothian & University of Edinburg, Edinburgh, UK
[110] Director, MRC Biostatistics Unit, Cambridge Institute of Public Health, Cambridge, UK
[111] Department of Physical Medicine and Rehabilitation, Oslo University Hospital/University of Oslo, Oslo, Norway
[112] Division of Orthopedics, Oslo University Hospital, Oslo, Norway
[113] Institue of Clinical Medicine, Faculty of Medicine, University of Oslo, Oslo, Norway
[114] Broad Institute, Cambridge MA Harvard Medical School, Boston MA, Massachusetts General Hospital, Boston MA, USA

[115] National Trauma Research Institute, The Alfred Hospital, Monash University, Melbourne, Victoria, Australia
[116] Department of Neurosurgery, Odense University Hospital, Odense, Denmark
[117] International Neurotrauma Research Organisation, Vienna, Austria
[118] Klinik für Neurochirurgie, Klinikum Ludwigsburg, Ludwigsburg, Germany
[119] Division of Biostatistics and Epidemiology, Department of Preventive Medicine, University of Debrecen, Debrecen, Hungary
[120] Department Health and Prevention, University Greifswald, Greifswald, Germany
[121] Department of Anaesthesiology and Intensive Care, AUVA Trauma Hospital, Salzburg, Austria
[122] Department of Neurology, Elisabeth-TweeSteden Ziekenhuis, Tilburg, the Netherlands
[123] Department of Neuroanesthesia and Neurointensive Care, Odense University Hospital, Odense, Denmark
[124] Department of Neuromedicine and Movement Science, Norwegian University of Science and Technology, NTNU, Trondheim, Norway
[125] Department of Physical Medicine and Rehabilitation, St.Olavs Hospital, Trondheim University Hospital, Trondheim, Norway
[126] Department of Neurosurgery, University of Pécs, Pécs, Hungary
[127] Division of Neuroscience Critical Care, John Hopkins University School of Medicine, Baltimore, USA
[128] Department of Neuropathology, Queen Elizabeth University Hospital and University of Glasgow, Glasgow, UK
[129] Dept. of Department of Biomedical Data Sciences, Leiden University Medical Center, Leiden, The Netherlands
[130] Department of Pathophysiology and Transplantation, Milan University, and Neuroscience ICU, Fondazione IRCCS Cà Granda Ospedale Maggiore Policlinico, Milano, Italy
[131] Department of Radiation Sciences, Biomedical Engineering, Umeå University, Umeå, Sweden
[132] Perioperative Services, Intensive Care Medicine and Pain Management, Turku University Hospital and University of Turku, Turku, Finland
[133] Department of Neurosurgery, Kaunas University of Health Sciences, Kaunas, Lithuania
[134] Intensive Care and Department of Pediatric Surgery, Erasmus Medical Center, Sophia Children's Hospital, Rotterdam, The Netherlands
[135] Department of Neurosurgery, Kings college London, London, UK
[136] Neurologie, Neurochirurgie und Psychiatrie, Charité – Universitätsmedizin Berlin, Berlin, Germany
[137] Department of Intensive Care Adults, Erasmus MC– University Medical Center Rotterdam, Rotterdam, the Netherlands
[138] icoMetrix NV, Leuven, Belgium
[139] Movement Science Group, Faculty of Health and Life Sciences, Oxford Brookes University, Oxford, UK
[140] Radiology Department, Antwerp University Hospital, Edegem, Belgium
[141] Director of Neurocritical Care, University of California, Los Angeles, USA
[142] Department of Neurosurgery, St.Olavs Hospital, Trondheim University Hospital, Trondheim, Norway
[143] Department of Emergency Medicine, University of Florida, Gainesville, Florida, USA
[144] Department of Neurosurgery, Charité – Universitätsmedizin Berlin, corporate member of Freie Universität Berlin, Humboldt-Universität zu Berlin, and Berlin Institute of Health, Berlin, Germany
[145] VTT Technical Research Centre, Tampere, Finland
[146] Section of Neurosurgery, Department of Surgery, Rady Faculty of Health Sciences, University of Manitoba, Winnipeg, MB, Canada

Additional CINTER-TBI participants and investigators:

Dr Deepak Agrawal[1], Dr Khursheed Alam Khan[2], Dr Sanjeev Bhoi[3], Dr Ashish Bindra[1], Dr Sachin Borkar[1], Dr Ajay Choudhary[4], Dr Madhur Choudhary[2], Dr Shivanand Gamanagatti[5], Dr Nand Kishore Gora[2], Dr Deepak Gupta[1], Dr Amit Gupta[6], Dr SS Kale[1], Dr Shweta Kedia[1], Dr Ashima Nehra[7], Dr Kokkula Praneeth[1], Dr Girija Rath[8], Dr GD Satyarthee[1], Dr Arul Selvi[9], Dr BS Sharma[1], Kaveri Sharma[1], Dr Rajeev Sharma[1], Dr Pankaj Kumar Singh[1], Dr VD Sinha[2,10], Dr Sumit Sinha[1], Dr Vivek Tandon[1]

[1] All India Institute of Medical Sciences, Department of Neurosurgery, New Delhi, India

[2] Sawai Man Singh Medical College, Department of Neurosurgery, Jaipur, Rajasthan, India

[3] All India Institute of Medical Sciences, Department of Emergency Medicine, New Delhi, India
[4] Dr Ram Manohar Lohia Hospital, Department of Neurosurgery, New Delhi, India
[5] All India Institute of Medical Sciences, Department of Radiology, New Delhi, India
[6] All India Institute of Medical Sciences, Department of Surgery, New Delhi, India
[7] All India Institute of Medical Sciences, Department of Neuropsychology, New Delhi, India
[8] All India Institute of Medical Sciences, Department of Neuroanaesthesia, New Delhi, India
[9] All India Institute of Medical Sciences, Department of Pathology and Lab Medicine, New Delhi, India
[10] Santokba Durlabhji Memorial Hospital cum Medical Research Institute, Department of Neurosurgery, Jaipur, Rajasthan, India
